# Fishbone-Associated Actinomycosis of the Anterior Cervical Space: A Diagnostic Dilemma

**DOI:** 10.1155/2010/282167

**Published:** 2010-11-22

**Authors:** Sang Kwon Lee, Mi Jeong Kim, Sun Young Kwon

**Affiliations:** ^1^Department of Radiology, Dongsan Medical Center, Keimyung University School of Medicine, Daegu 700-712, Republic of Korea; ^2^Department of Pathology, Dongsan Medical Center, Keimyung University School of Medicine, Daegu 700-712, Republic of Korea

## Abstract

We report the imaging and pathologic findings of fishbone-associated actinomycosis of the anterior cervical space in a 57-year-old man, misdiagnosed preoperatively as a malignancy originating from thyroglossal duct cyst. CT revealed an enhancing mass containing a small abscess pocket and two sharp linear calcifications within it, which infiltrated into the strap muscle. Pathologic examination demonstrated two fishbones within the actinomycotic abscess. Fishbone-associated actinomycosis should be considered when a cervical mass contains sharp linear calcifications.

## 1. Introduction

Fishbone foreign bodies are usually found in the pharynx, and most of them can be easily removed. Migration out of the pharynx is an unusual event, and most of them penetrate into the retropharyngeal or prevertebral space, and rarely into the thyroid gland or lateral neck [1, 2]. Migration of fishbone into the anterior cervical space is thought to be quite unusual, as the relatively hard larynx is located anterior to the pharynx.

Actinomycosis is a chronic, suppurative, fibrosing infection caused by filamentous, gram-positive anaerobic bacteria that are normal constituents of the oral flora. *Actinomyces israelii* is the most common agent in human actinomycosis. Cervicofacial involvement is the most frequent manifestation of actinomycosis, accounting for approximately 50 percent of all cases, while central nervous system, thoracic, abdominal, and pelvic actinomycosis occur less frequently [3, 4]. Predisposing factors include poor oral hygiene; tooth decay or abscess or periodontal disease; gingivitis and gingival trauma; diabetes mellitus; immunosuppression; malnutrition; local tissue damage. Actinomycosis is usually diagnosed by culturing the organism, which requires extended anaerobic or microaerophilic incubation, and by a biopsy specimen from different tissue levels. The histological examination demonstrates organisms in a filamentous form with branching hyphae and characteristic sulfur granules. The treatment of choice is a prolonged course of high-dose penicillin coupled with appropriate surgical intervention for complicated cases. The lesion of cervicofacial actinomycosis can mimic many other diseases, including neoplasms of the head and neck. Fishbone-associated actinomycosis of the anterior cervical space has not appeared in the literature, while cases of foreign body-associated actinomycosis elsewhere have been reported [5, 6].

We report here a case of fishbone-associated actinomycosis of the anterior cervical space in a 57-year-old man, misdiagnosed preoperatively as a malignancy originating from thyroglossal duct cyst (TGDC), along with its imaging and pathologic features.

## 2. Case History

**Figure 1:**
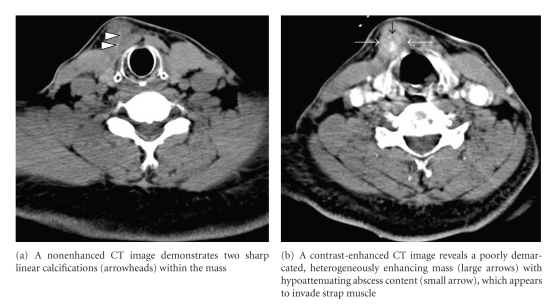
CT features of fishbone-associated actinomycosis of the right anterior cervical space.

**Figure 2:**
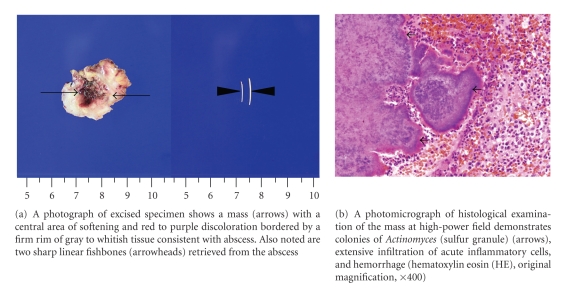
Gross and microscopic features of fishbone-associated actinomycosis.

A 57-year-old man presented with a painless anterior neck mass which had developed six months prior and had gradually enlarged over that period. Two months prior, he had undergone CT examination and fine-needle aspiration biopsy (FNAB) for progressively enlarging anterior neck mass at other hospitals. He was afebrile. On physical examination, a nontender mass of approximately 3 cm in size was found in the right anterior neck near the midline. There was no associated skin discoloration or cervical lymphadenopathy. No abnormal findings were seen in the oral cavity or pharynx on direct visualization or fiberscopic examination. All laboratory tests were within the normal limit except slightly elevated alanine transaminase (ALT) of 56 U/L (normal range: 5–44 U/L). Nonenhanced CT (NECT) (Figure 1(a)) demonstrated a poorly demarcated mass with two sharp linear calcifications, and contrast-enhanced CT (CECT) (Figure 1(b)) showed a heterogeneously enhancing mass with a small abscess pocket in the right anterior cervical space near the midline, between the platysma and strap muscles. The mass appeared to invade into the right strap muscle (Figure 1(b)). On the basis of clinical and imaging findings, a malignancy originating from the TGDC was suggested to be a possible diagnosis. He underwent surgical excision of the mass. At operation, an about 3 × 3 cm sized irregular mass with solid and cystic components that adhered severely to the adjacent structures was noted (Figure 2(a)). Two sharp linear fishbone foreign bodies were identified within the mass (Figure 2(a)). Pathologic findings of the excised specimen were consistent with fishbone-associated actinomycosis (Figure 2(b)). Postoperative course was uneventful without recurrence until 30 months after operation.

## 3. Discussion

One of the uncommon complications of accidentally ingested foreign bodies is migration, which has the potential to cause morbidity and mortality. Fishbones are common cause of migrating foreign bodies [7]. Although, the timing of ingestion of fishbones and the route of penetration to the anterior cervical space have not been verified, migration of ingested fishbones from the hypopharynx or cervical esophagus was supposed to be the predisposing condition for actinomycosis. Intraluminal and extraluminal penetrating foreign bodies may remain quiescent for years before presenting a complication. Our patient presented with anterior neck mass, but he was afebrile and felt painless. The mass was nontender without skin discoloration. These clinical manifestations presented diagnostic dilemma. The only diagnostic clue was sharp, linear calcifications within the mass. We had noticed these sharp linear calcifications on NECT at first glance. However, we could not imagine that the linear calcifications were accidentally ingested fishbones because they were too far away from the pharynx or esophagus, which is believed to be a site of primary impaction of the fishbones, to be migrated to the final destination of the anterior cervical space.

Migrating foreign bodies outside of the pharynx or esophagus are often complicated by severe infection or abscess formation in the deep cervical spaces. Thus, identification and removal of the body as soon as possible is important. Actinomycosis of the head and neck has long been known as the “great masquerader” because it can mimic other benign or malignant processes, thus, making definitive diagnosis difficult [8]. CT features of actinomycosis of the neck consist of a thick-walled enhancing mass with a low-attenuation center [9]. Our case represents the first case of fishbone-associated actinomycosis abscess occurring at the anterior cervical space, which mimicked malignancy originating from TGDC. The findings at FNAB which had been performed at other hospitals suggested TGDC. We initially suggested papillary carcinoma containing calcifications originating from the TGDC because the mass was located at the right anterior cervical space near the midline, and it appeared to invade strap muscle. We retrospectively speculated that the sharp linear calcifications might be a useful diagnostic clue for migrated fishbones even if they were located far away from the pharynx or esophagus.

In summary, the clinician should suspect a fishbone-associated actinomycosis when a patient has a cervical mass containing sharp linear calcifications on CT. Although most cases of cervical actinomycosis are of odontogenic origin, primary infections have been reported in the head and neck regions. The indolent course resembling that of malignancy originating from TGDC can confound early diagnosis. Appropriate steps to obtain an adequate specimen for histopathologic examination and culture are required for definitive diagnosis of actinomycosis.
